# Supplementary figures and images for: Rhythmidia: A modern tool for circadian period analysis of filamentous fungi
Source: PLoS Comput Biol. 2024 Aug 5;20(8):e1012167. doi: 10.1371/journal.pcbi.1012167 (PMC11326708; doi:10.1371/journal.pcbi.1012167)

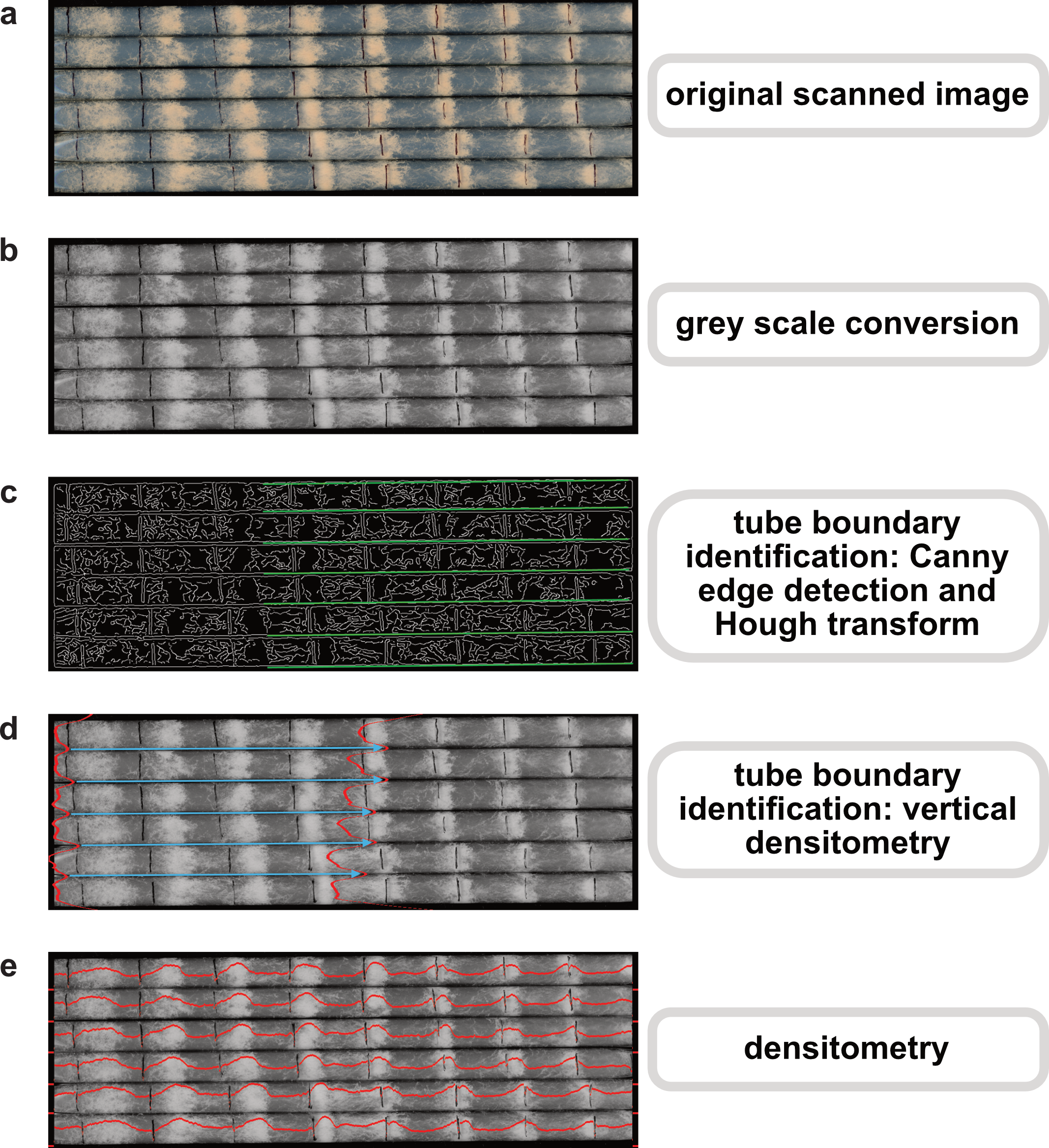

Supplement: S1 Fig — a. Original scan of 6-pack of representative race tubes of strain 328–4 to depict algorithmic feature identification. b. Representative 6-pack post-Rhythmidia greyscale conversion. c. Depiction of detected Canny edges in representative race tube image to identify lines demarcating individual race tubes (green lines). d. Vertical bilateral densitometry (red traces) of a race tube image for identification of lines demarcating individual race tubes. Blue arrows indicate horizontal tube demarcation lines drawn between corresponding density minima. e. Raw densitometry of each race tube overlaid upon the corresponding race tube image. (TIFF) [file pcbi.1012167.s001.tiff]

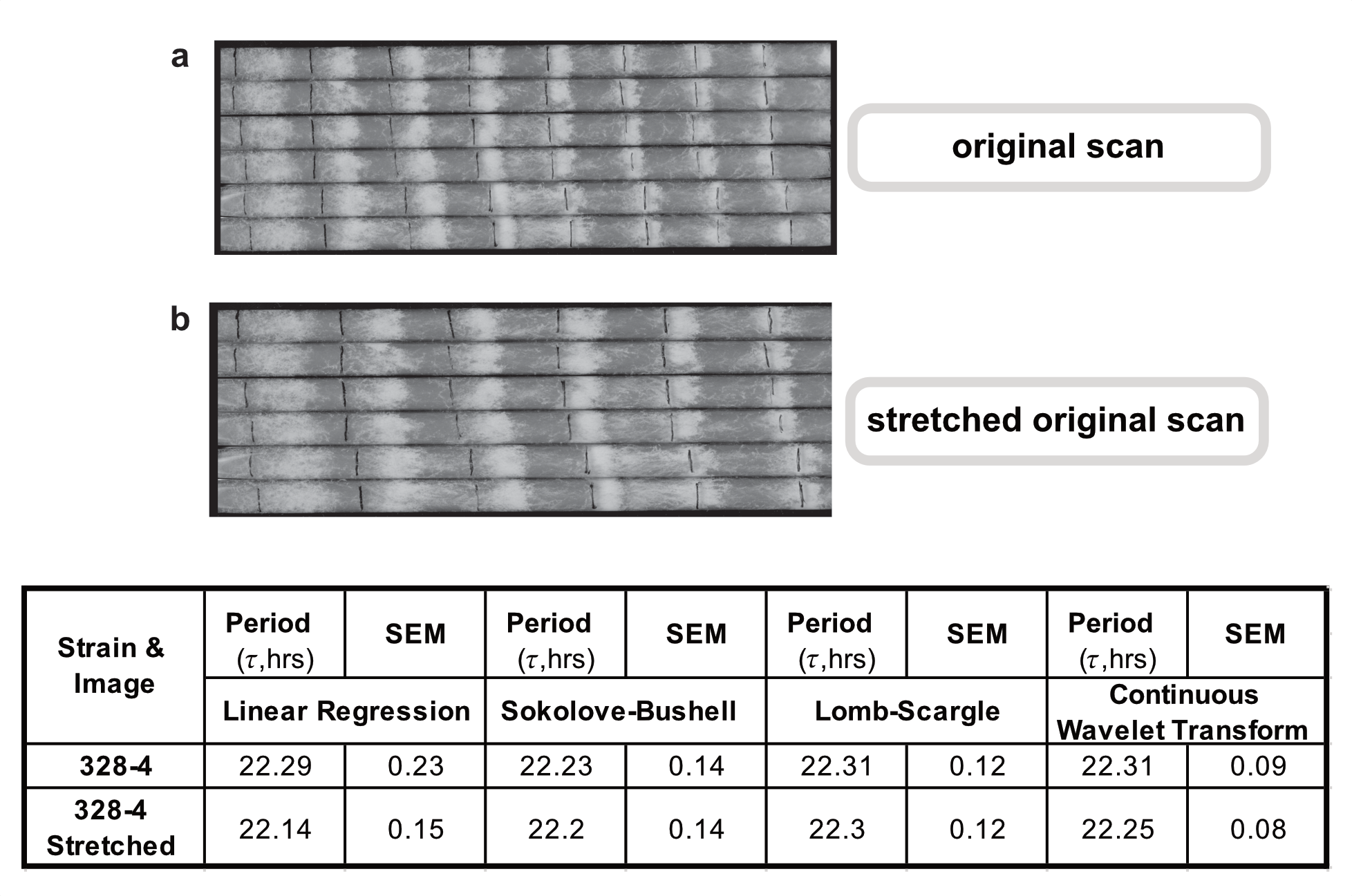

Supplement: S2 Fig — Rhythmidia greyscale image output (a.) of analyzed image, as well as of cropped version of same image after programmatic resizing (b., stretching). The table below displays calculated periods (τ) in hours for both versions of the image, calculated using the linear regression, Sokolove-Bushell periodogram, Lomb-Scargle periodogram, and continuous wavelet transform. For each method, the period is calculated for each of six tubes, and these calculations are used to calculate the arithmetic mean and the standard error of the mean (SEM) for n = 6 tubes. (TIFF) [file pcbi.1012167.s002.tiff]

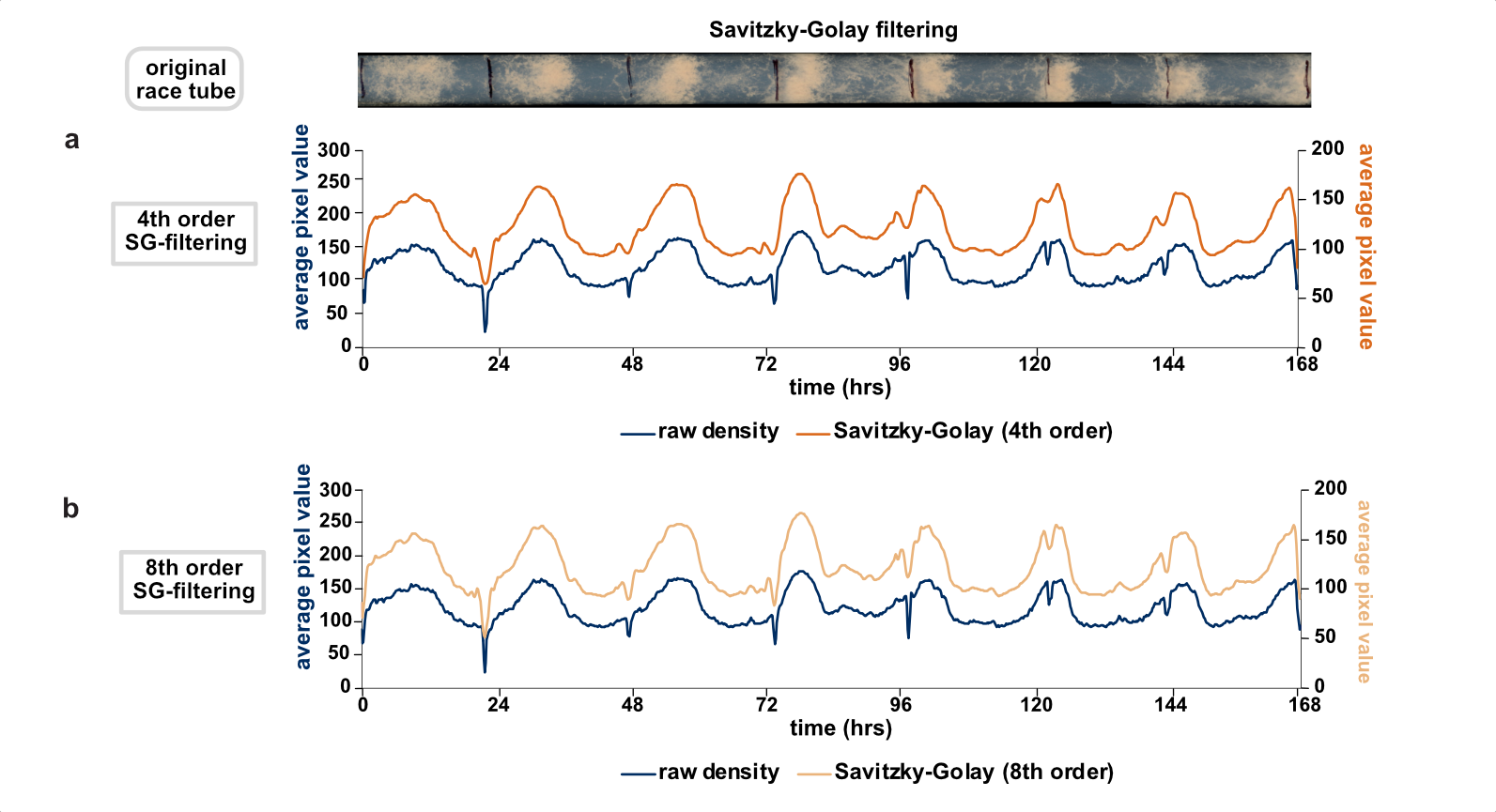

Supplement: S3 Fig — A single race tube image, aligned with representations of the effects of Savitzky-Golay filtering. Each plot compares the filtered to the raw densitometry profile of a race tube using fourth-order (a.) and eighth-order (b.) fitting. Densitometry is assessed as an average pixel value, with higher values corresponding to brighter pixels in a greyscale image. (TIFF) [file pcbi.1012167.s003.tiff]

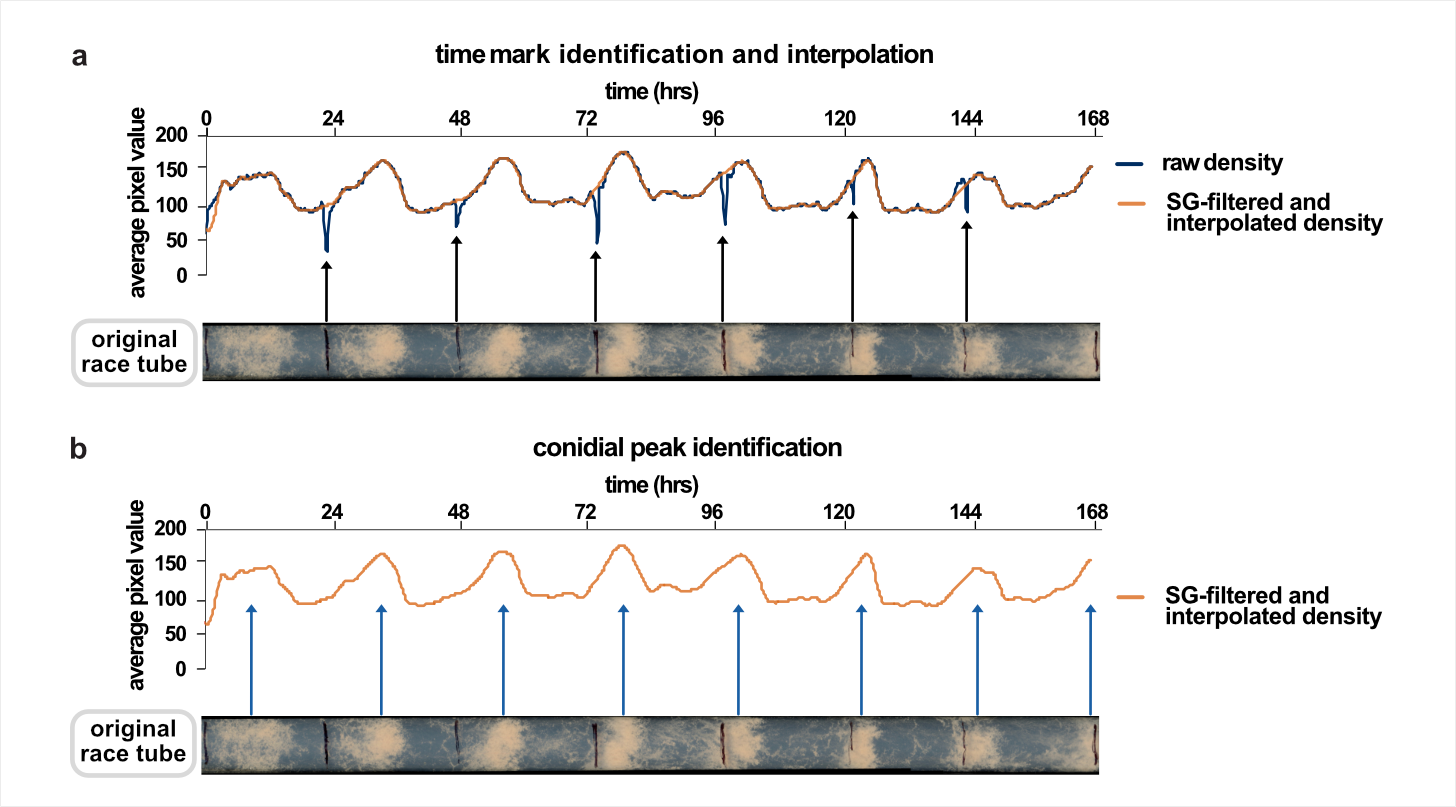

Supplement: S4 Fig — a. Comparison of raw densitometry overlaid with smoothed, interpolated densitometry of a single race tube, aligned with the original image of the tube. Black arrows connect time marks in the image to corresponding local minima artifacts in the raw data. b. Smoothed, interpolated densitometry of a single race tube, aligned with the original image of the tube. Blue arrows indicate sites of conidial banding (peaks) in the image to corresponding local maxima in the plot. (TIFF) [file pcbi.1012167.s004.tiff]

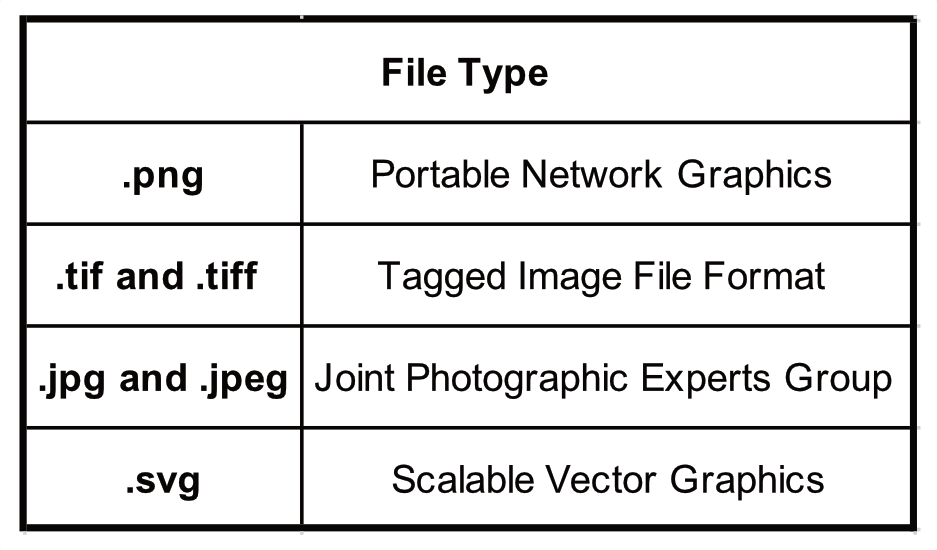

Supplement: S1 Table — (TIFF) [file pcbi.1012167.s005.tiff]
